# Tissue-specific T cell profiles in *Mycobacterium tuberculosis* uninfected IGRA negative and positive individuals

**DOI:** 10.3389/ftubr.2025.1535945

**Published:** 2025-03-06

**Authors:** Gift Ahimbisibwe, Marjorie Nakibuule, Marvin Martin Ssejjoba, Claire Precious Bisoboka, Feddy Gift Akello, Marvin Joven Turyasingura, Rose Mulwana, Josephine Nabulime, Febronius Babirye, Musana Abdusalaamu Kizito, Hervé Monka Lekuya, Akello Suzan Adakun, Daisy Nalumansi, Stella Muryasingura, Robert Lukande, Joseph Baruch Baluku, Irene Andia Biraro, Stephen Cose

**Affiliations:** ^1^MRC/UVRI and LSHTM Uganda Research Unit, Entebbe, Uganda; ^2^Mulago National Referral Hospital, Kampala, Uganda; ^3^Department of Surgery, College of Health Sciences, Makerere University, Kampala, Uganda; ^4^Department of Pathology, Makerere University, Kampala, Uganda; ^5^Division of Pulmonology, Kiruddu National Referral Hospital, Kampala, Uganda; ^6^Department of Internal Medicine, Makerere University, Kampala, Uganda

**Keywords:** IGRA, *Mycobacterium tuberculosis*, tissues, T cells, TRMs

## Abstract

**Introduction:**

Interferon-gamma release assays (IGRAs), such as the T-SPOT.*TB* and QuantiFERON-TB Gold, are commonly used to detect immune responses to Mycobacterium tuberculosis (M.tb) and identify latent TB infection. However, their role in reflecting immune dynamics within tissues, especially in the absence of active disease, remains unclear.

**Methods:**

Post-mortem tissues, including lung, lymph nodes, spleen, and bronchoalveolar lavage, were collected from apparently healthy, HIV-negative road traffic accident victims. M.tb infection was ruled out using liquid MGIT culture, while M.tb exposure history was assessed with the TSPOT.*TB* assay. T and B cell phenotyping was performed using a 29-color flow cytometry panel, with data analyzed in FlowJo and GraphPad Prism.

**Results:**

Of the 52 individuals recruited, 48% were IGRA-positive (TSPOT+). Using a 29-color flow cytometry panel, we analyzed T and B cell populations across various tissues. We observed similar overall frequencies of CD3, CD4, CD8, and CD19 cells, as well as memory T and B cell subsets defined by CCR7/CD45RA and IgD/CD27 between TSPOT+ and TSPOT individuals. Notably, in the lungs, TSPOT+ individuals exhibited a higher frequency of CD4+ tissue-resident memory (TRM) T cells, along with increased expression of KLRG1, a marker of terminal differentiation, on mature CD4+CD27 T cells. This phenotype was specific to CD4 T cells in the lungs, highlighting the known role of CD4 T cells in TB immunity and their localization to the primary site of infection.

**Discussion:**

Our findings suggest that IGRA positivity, while indicating immune memory, may also be associated with highly differentiated CD4 T cells in tissue-specific compartments, particularly in the lungs. These localized immune changes raise important questions about the long-term effects of chronic immune engagement following repeated M.tb exposure in endemic settings. Further research is needed to assess the clinical implications of these findings, including their impact on susceptibility to future infections or disease progression.

## Introduction

Interferon-gamma release assays (IGRAs), such as the T-SPOT.*TB* and QuantiFERON-TB Gold, are *in vitro* enzyme-linked immunosorbent spot (ELISPOT) tests that identify *Mycobacterium tuberculosis (M.tb)*-specific T cells in blood samples (1). These assays provide a contemporary alternative to the traditional tuberculin skin test (TST), which measures delayed hypersensitivity to injected tuberculin (2). Unlike TSTs, IGRAs directly quantify the release of interferon-gamma (IFN-γ) from peripheral T cells upon exposure to *M.tb*-specific antigens, in particularly early secreted antigenic target 6 (ESAT-6) and culture filtrate protein 10 (CFP-10), both absent in Bacillus Calmette-Guérin (BCG) and most environmental mycobacteria (3, 4).

Historically, individuals with positive IGRA or TST results were labeled as “latent TB infected” (5, 6), implying they harbored a quiescent *M.tb* infection that could progress to active disease, necessitating prophylactic treatment. The assumption here is that the result of treatment should be a negative IGRA test which is now known not to be the case; an IGRA result can remain positive even after curative TB treatment, due to the presence of memory T cells, the founding principle upon which the IGRA is based. These memory cells can outlive the infection by at least 9 years (7, 8). These findings have led to the idea that examining the diversity of T cell phenotypes may be more helpful in distinguishing between latent and active TB, as well predicting the likelihood of progression from one state to another.

Most TB studies focus on differences between IGRA positive individuals and those with active TB disease, often highlighting differences in T cell activation and differentiation profiles. For example, studies report lower expression of the markers CD27, CD127, CCR7, and CD45RA in individuals with active TB compared to IGRA positive individuals (9–11). Others have shown increased expression of the activation markers HLA-DR and CD38 in active TB (12). However, few studies have compared IGRA positive individuals directly with IGRA negative individuals to understand what T-SPOT positivity or negativity means in relation to *M.tb* immune recall.

One study investigating household contacts of TB patients found that IGRA positive individuals exhibited elevated levels of CD4+CD25+CD127- regulatory T cells (Tregs) in bronchoalveolar lavage (BAL) fluid compared to their IGRA negative counterparts (13), suggesting enhanced local immune regulation in response to *M.tb* exposure. However, it remains unclear whether IGRA negative individuals are at lower risk of developing TB or if their immune profiles indicate a different form of immune protection or vulnerability.

The traditional dichotomy of latent and active TB has been increasingly challenged. Current research suggests a spectrum of TB infection that encompasses six stages: uninfected, TB infection, incipient TB, subclinical TB without symptoms, subclinical TB with unrecognized symptoms, and overt TB disease (14). IGRA positivity can be detected across these various stages, from early immune recognition of *M.tb* to subclinical or resolved infections (15). As a result, studies may have inadvertently grouped together different latent TB states, each with its own unique immune characteristics. A better understanding IGRA positivity in the absence of active infection could offer valuable insights into successful *M.tb* clearance.

TB disease is largely a disease of the lung. The interpretation of peripheral IGRA positivity might be more informative if we can study it in the context of the extent of *M.tb* pathology in the lung. In the absence of infection, a positive IGRA test confirms immunological memory to a previous infection. Animal models have shown that exposure to *M.tb* drives IGRA conversion (16) and subsequent generation of more protective tissue resident cells within the lung (17). We do not know if this is the case in humans.

To understand how peripheral IGRA positivity, likely representing immune recall, translates within tissues, we compared T and B cell phenotypes in *M.tb* culture-negative, TSPOT positive and negative individuals across various tissues collected in a postmortem study (18).

## Materials and methods

### Ethics

This study obtained ethical approval from five ethics bodies: the Makerere University School of Biomedical Sciences Research and Ethics Committee (SBS-REC-721), the Mulago National Referral Hospital Ethics Committee (MHREC 1849), the Kiruddu National Referral Hospital School of Biochemical Research and Ethics Committee (CRD/ADMIN/120/1), the Uganda National Council of Science and Technology Ethics Committee (HS703ES), and the London School of Hygiene and Tropical Medicine Ethics Committee (22,922).

### Consent

Trained grief counselors were employed to sensitively obtain informed consent from the next-of-kin (NoK) as described by Uganda Law. The NoK gave consent for a full postmortem, for donation of tissues for medical research and future use, and for access to previous medical records. A case record form was used by the counselors to collect reasons for consent or decline from the NoK.

### Participants

We recruited deceased road traffic accident subjects from the Surgical Emergency Unit (SEU), Mulago National Referral Hospital Kampala, Uganda, between January 2021 and June 2022. Only subjects above 18 years of age whose NoKs consented into the study were recruited into the study. Recruited subjects were otherwise healthy at the time of death, with no obvious underlying morbidity or chest involvement at the time of death. Patient demographics are described in Supplementary Table 1.

### Tissues and sample collection

A full postmortem was performed by the study pathologist on all study subjects to establish the cause of death and identify any underlying conditions that may have been missed during routine clinical and laboratory examination. Tissue samples, including lung, lymph nodes draining the lung (Hilar Lymph Nodes—HLNs), spleen, peripheral lymph nodes (Iliac and inguinal lymph nodes; PLNs) and blood were collected. Solid tissues were placed in 50 ml tubes with 20% FBS in RPMI medium. Bronchoalveolar lavage (BAL) washing of both the left and right lungs were performed with PBS. Arterial blood was collected from the carotid artery into heparin tubes; arteries were considered instead of veins due to regular venous collapse after death. Sample tubes were tightly capped, placed in a rack in a sealable cool box and transported to the BSL3 laboratory at the MRC/UVRI and LSHTM Uganda Research Unit at room temperature. All sample processing was performed under BSL3 conditions.

### PBMC isolation

Heparinised blood was diluted with RPMI media and layered manually on FicollPaque PLUS media. PBMCs were then isolated by density centrifugation. The buffy coat was harvested and centrifuged to obtain a cell pellet. Red blood cells in the pellet were lysed, the cells washed and then counted on an automated cell counter (TC20; Biorad).

### Cell isolation from tissues

#### Lung

Cells were obtained from lung tissue by enzymatic digestion using collagenase D (1 mg/ml) and DNase I (1 g/ml; hereafter referred to as “the enzyme mixture”) and physical disintegration using the gentleMACS Octo Dissociator (Milteyni Biotech) as described previously (19). Briefly, the lungs were cut into small pieces using fine scissors and forceps in a sterile petri dish, placed in gentleMACS purple C tubes with the enzyme mixture, loaded on the gentleMACS Octo Dissociator and run using Lung Program 1. The tubes were then incubated in a CO_2_ incubator for 25 min and then run using Lung Program 2. One milliliter of smashed tissue was taken off to perform MGIT culture. The sample was then filtered through a 70 μm filter followed by a 40 μm filter, centrifuged at 600 rcf for 5 min to obtain a cell pellet. Residual red blood cells were lysed using ACK lysis buffer, leaving behind a pure black cell pellet. We assume the cell pellet from the draining lungs was black due to a lifetime exposure to carbon residues as a result of inhalation of carbon fumes from vehicles, firewood, and charcoal smoke (20). The cells were washed in RPMI and counted using an automated TC20 cell counter (Biorad).

#### Lymph nodes

Lymph nodes were first cleaned by teasing the lymph node tissue from surrounding fat using forceps and scissors, cut into small pieces using fine scissors and forceps in a sterile petri dish and mixed with RPMI media. The resultant mixture was then filtered through a 70 μm filter followed by a 40 μm filter, then centrifuged at 600 rcf for 5 min to obtain a cell pellet. Red blood cells were lysed using ACK lysis buffer. The resultant pellet was washed with RPMI and counted using an automated TC20 cell counter.

#### Spleen

Spleen sections were chopped into small pieces using fine scissors and forceps in a sterile petri dish and mixed with RPMI media. The resultant mixture was then filtered through a 70 μm filter and centrifuged at 600 rcf for 5 min to obtain a cell pellet. The pellet was then reconstituted with RPMI and layered manually onto FicollPaque PLUS media. Spleen mononuclear cells were then obtained by density centrifugation. The buffy coat was harvested and centrifuged to obtain a cell pellet. Residual red blood cells in the pellet were lysed using ACK lysis buffer, and the cells then washed and counted using an automated TC20 cell counter.

#### Bronchoalveolar lavage fluid

BAL was collected in 50 ml PBS, using a standard BAL procedure, but washing both lungs and deeper airways than would normally be performed for a living subject. BAL fluid was filtered through a 70 μm filter followed by a 40 μm filter, then centrifuged at 600 rcf for 5 min to obtain a cell pellet. Red blood cells were lysed using ACK lysis buffer. The resultant pellet was washed with RPMI and counted using an automated TC20 cell counter.

### T-SPOT^®^.*TB* assay

This assay was performed using the T-SPOT^®^.*TB* kit (TB.300, Oxford Immunotec) to enumerate individual TB-specific activated effector T cells producing IFNγ. Samples were run in duplicate. The procedure was performed as per insert, except for incubation time (36 h) and media (RPMI plus 10% FBS). Extensive testing showed that this time and media yielded best results from our deceased subjects, above that of the recommended protocol for live venous blood. Resultant spots were read using an ELISPOT reader (AID *i*Spot ELR08IFL), and were interpreted as per insert.

### BACTEC MGIT 960 for recovery of *M.tb*

One milliliter of smashed tissue or BAL was decontamination with an equal volume of mycoprep (Cat. No. 240862, 240863) for 30 min while vortexing every 5 min. Positive and negative controls of known time to positivity and bacterial load were included to control for the decontamination process. Sterile Phosphate Buffered Saline (PBS) was then added to stop the reaction followed by a 3,500 g spin for 15 min. The sediment was then inoculated into the MGIT tubes with BBL™ MGIT™ PANTA™ Antibiotic Mixture (245,114) to minimize contamination and enhance *M.tb* growth. MGIT tubes were then incubated in the BD BACTEC™ MGIT™ Automated Mycobacterial Detection System for 42 days. Positive growths were confirmed by positive Ziehl-Neelsen (ZN) staining and the MPT64 antigen assay.

### Cell phenotypic analysis by flow cytometry

**Figure 1:**
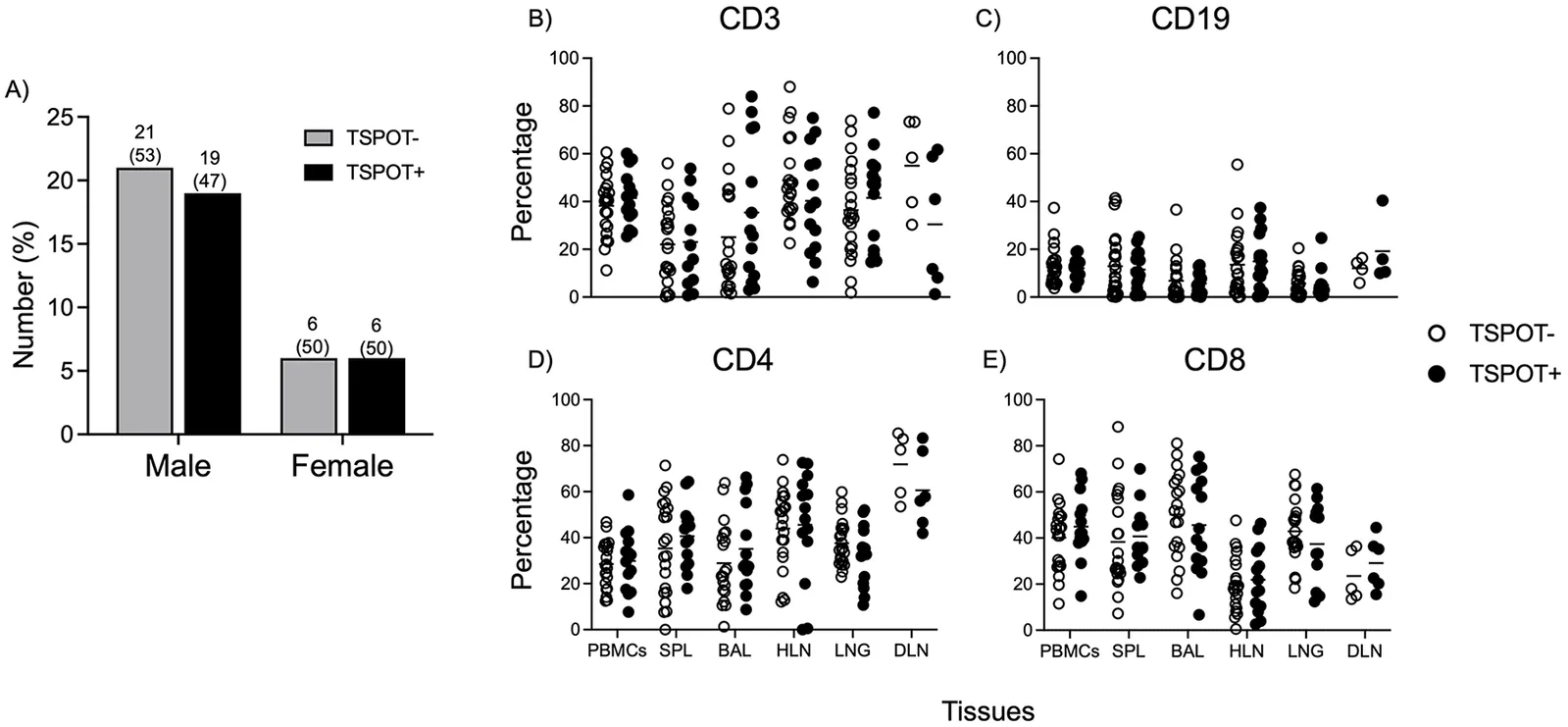
The frequencies of CD3, CD4, CD8, and CD19 cell subsets are similar between TSPOT positive and negative individuals, and across all tissues. PBMCs were processed from arterial blood after death, and the TSPOT. *TB* assay used to assess prevalence **(A)**. Data shows frequencies of CD3 **(B)**, CD19 **(C)**, CD4 **(D)**, and CD8 **(E)** cells in the Blood, Spleen, Bronchoalveolar lavage (BAL), Hilar lung draining lymph nodes (HLN), Lung tissue and distal (iliac and/or inguinal) lymph node (DLN). No differences were observed between TSPOT positive and negative individuals. A comparison between different tissues is available in Supplementary Figure 1. Data was from all subjects. The Mann Whitney test was used to compare medians between the two groups, with Bonferroni correction applied to account for multiple comparisons. Statistical significance was defined as a corrected *p* < 0.05, indicated by lines above each graph.

**Figure 2:**
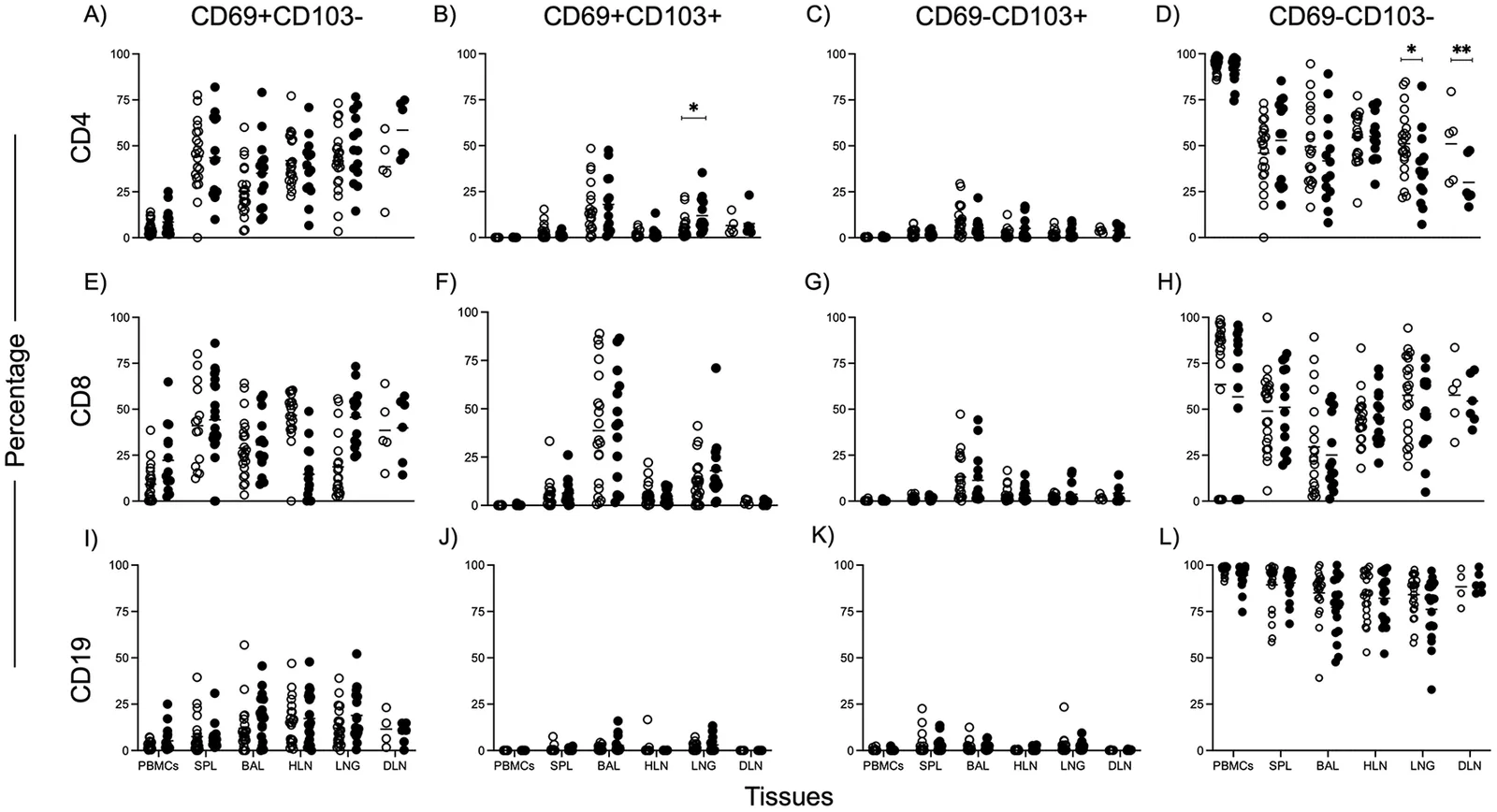
Higher CD4 Tissue Resident Memory T cell frequencies in the lungs of TSPOT positive individuals. CD69 and CD103 markers were used to identify Tissue Resident Memory subsets. Data shows frequencies of subset (y-axis) in tissues (x-axis) of TSPOT positive and negative individuals. Data was from all subjects. The Mann Whitney test was used to compare medians between the two groups, with Bonferroni correction applied to account for multiple comparisons. Statistical significance was defined as a corrected *p* < 0.05, indicated by lines above each graph. Plots showing CD69+CD103 **(A, E, I)**, CD69+CD103+ **(B, F, J)**, CD69−CD103+ **(C, G, K)**, CD69−CD103 **(D, H, L)** for CD4 T cells **(A–D)**, CD8 T cells **(E–H)**, and CD19 B cells **(I–L)**.

A 29-color antibody T and B cell panel was used to phenotype cells isolated from each of the tissues. The gating strategy for PBMCs and tissues are shown in Figures 1, 2, respectively. Antibodies used were CD3 (PACIFIC BLUE HIT3a BioLegend), CD38 (BV510 HIT2 Biolegend), CCR7 (BV605 G043H7 Biolegend), PD-1 (BV650 NAT105 Biolegend), CD69 (BV711 FN50 Biolegend), CD28 (BV750 CD28.2 Biolegend), CD27 (BV785 O323 Biolegend), CD8 (SPARK BLUE 550SK1 Biolegend), FCLR4 (PE413D12 Biolegend), CD127 (SPARK YG581 A019B5 Biolegend), CD4 (PECY5 RPA-T4 Biolegend), CD57 (PECY7 HNK-1 Biolegend), IgG (APC M1310G05 Biolegend), KLRG1 (ALEXAFLUOR 647 SA231A2 Biolegend), CD21 (ALEXAFLUOR 700 Bu32 Biolegend), HLADR (APC-FIRE 810 I243 Biolegend), LIVE/DEAD (ZOMBIE UV Biolegend), CD103 (BUV395 Ber ACT8 BD), CD25 (BUV496 M-A251 BD), CD196 (BUV563 11A9 BD), CD278 (BUV661 DX29 BD), IgM (BUV737 UCH-B1 BD), CD45RA (BUV805 HI100 BD), CD5 (BV421 UCHT2 BioLegend), CD24 (FITC eBioSN3 eBioscience), CD183 (BB700 1C6/CXCR3 BD), CD10 (PerCP efluor710 SN5c Life technologies), IgD (PE-Dazzle594 IA6-2 BD) and CD19 (APC-H7 SJ25C1 BD).

Viability staining was performed first with the fixable viability dye, zombie UV, for 20 min in the dark at room temperature. Cells were washed and resuspended in 100 μl of surface antibody cocktail for 20 min in the dark at 4°C, after which they were washed to remove excess antibody. Cells were acquired on the Cytek Aurora Spectral Analyser. All flow cytometry data was analyzed using FlowJo version 10.8.1. Compensation controls to remove spectral overlap and Fluorescence Minus One (FMO) controls were used to establish gates. Representative gating strategies for PBMC and lung samples are shown in Figures 1, 2, respectively.

### Statistical analysis

GraphPad Prism Version 9 was used for statistical analysis and figure generation. Excel files with percentage expression of cell types were exported from FlowJo and imported into GraphPad Prism. The Mann Whitney test, a non-parametric method was used for comparing medians between the two groups. To account for multiple comparisons, a Bonferroni correction was applied. Statistical significance was defined as the corrected *p* < 0.05.

## Results

To gain a better understanding of what peripheral IGRA positivity represents in tissues, and to explore whether there are tissue-specific differences between IGRA+ and IGRA– individuals, we leveraged an ongoing TB Postmortem study (18). The primary goal was to investigate how the recall response measured by IGRA positivity translates into immune cell behavior in various tissues.

We recruited 52 HIV-negative deceased subjects from the Surgical Emergency Unit of MNRH. Full postmortem examination confirmed traumatic injury as the cause of death in most cases. All participants were otherwise healthy, with no known clinical conditions and normal parameters. There was a male predominance (Supplementary Table 1), which aligns with the high proportion of male motorists involved in accidents in Uganda. Arterial blood and tissues were collected from these individuals, with informed consent from the next-of-kin (NoK).

To ensure that none of the participants had active *M. tuberculosis (M.tb)* infection, a MGIT culture was performed on all tissues. The TSPOT.*TB* assay was used on PBMCs from arterial blood to differentiate individuals into IGRA+ (TSPOT+) or IGRA– (TSPOT–). Out of the 52 subjects recruited, 25 (48%) were TSPOT positive (Figure 1A), a prevalence consistent with previous reports from Uganda (21). As a result, we focused on analyzing immune cells present in the tissues of individuals with a recall immune response to *M.tb* as defined by the T-SPOT.TB assay.

Using a 29-color panel, we examined T and B cell phenotypes across different tissues in the two groups. The gating strategy is shown in Supplementary Figure 1 (PBMCs) and Supplementary Figure 2 (other tissues). Total frequencies of CD3, CD4, CD8, and CD19 cells were similar between TSPOT+ and TSPOT– individuals across all tissues (Figures 1A–E). There were no notable differences in CD45RA/CCR7-defined memory T cell subsets for CD4 or CD8 T cells (Supplementary Figure 3), nor in IgD/CD27 memory subsets for CD19 B cells between the two groups (Supplementary Figure 4), suggesting that the overall memory T and B cell landscapes are similar in both IGRA-positive and negative individuals in the absence of *M.tb* infection.

Given the importance of tissue-resident memory T cells (TRMs) in long-term immune surveillance, we focused on analyzing TRMs, defined by CD69 and CD103 expression, for CD4, CD8, and CD19 cells (Figure 2). Notably, we found a higher frequency of CD4+/CD69+/CD103+ TRMs in the lungs of TSPOT positive individuals compared to TSPOT negative individuals (Figure 2B), indicating a greater degree of immune cell retention, potentially reflecting an enhanced immune response to prior *M.tb* exposure in this cell subset. No significant differences in either CD8 or CD19 TRM frequencies (Figures 2E–L) were observed in the lungs or other tissues between the two groups.

**Figure 3:**
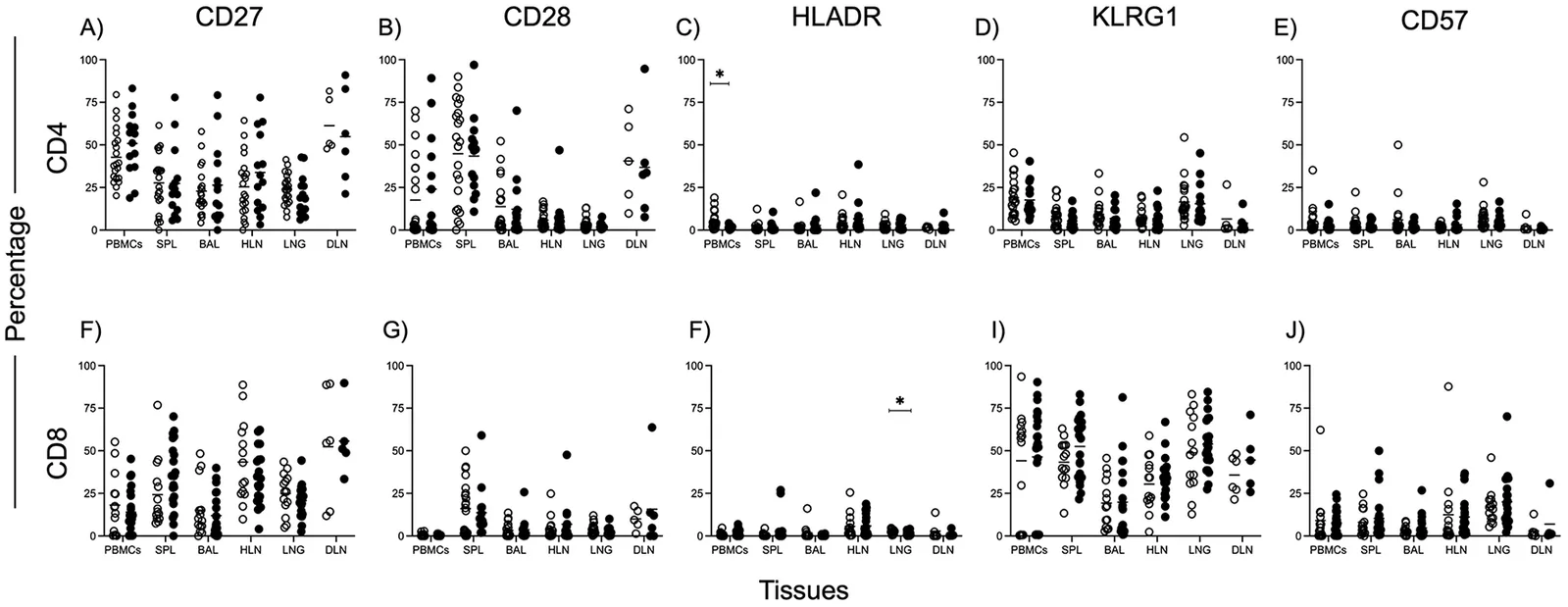
Activation, differentiation, and senescence of CD4 and CD8 T cells in different tissues. Phenotypical markers were used to assess activation [CD27—**(A,F)**, CD28—**(B,G)**, HLADR—**(C,F)**], terminal differentiation [KLRG1—**(D,I)**] and senescence [CD57 **(E,J)**], of CD4 **(A–E)** and CD8 **(F–J)** gated T cells. Data was from all subjects. The Mann Whitney test was used to compare medians between the two groups, with Bonferroni correction applied to account for multiple comparisons. Statistical significance was defined as a corrected *p* < 0.05, indicated by lines above each graph.

We also examined markers of T cell activation (HLA-DR, CD28), differentiation (CD27, KLRG1), and senescence (CD57) across tissues (Figures 3A–J). Interestingly, we observed higher HLA-DR expression on CD4+ T cells in the peripheral blood of TSPOT negative individuals (Figure 3C), while higher HLA-DR expression was seen on CD8 T cells in the lungs of TSPOT positive individuals (Figure 3F). These findings suggest distinct patterns of immune activation, likely reflecting chronic immune responses to prior *M.tb* exposure in IGRA-positive individuals.

**Figure 4:**
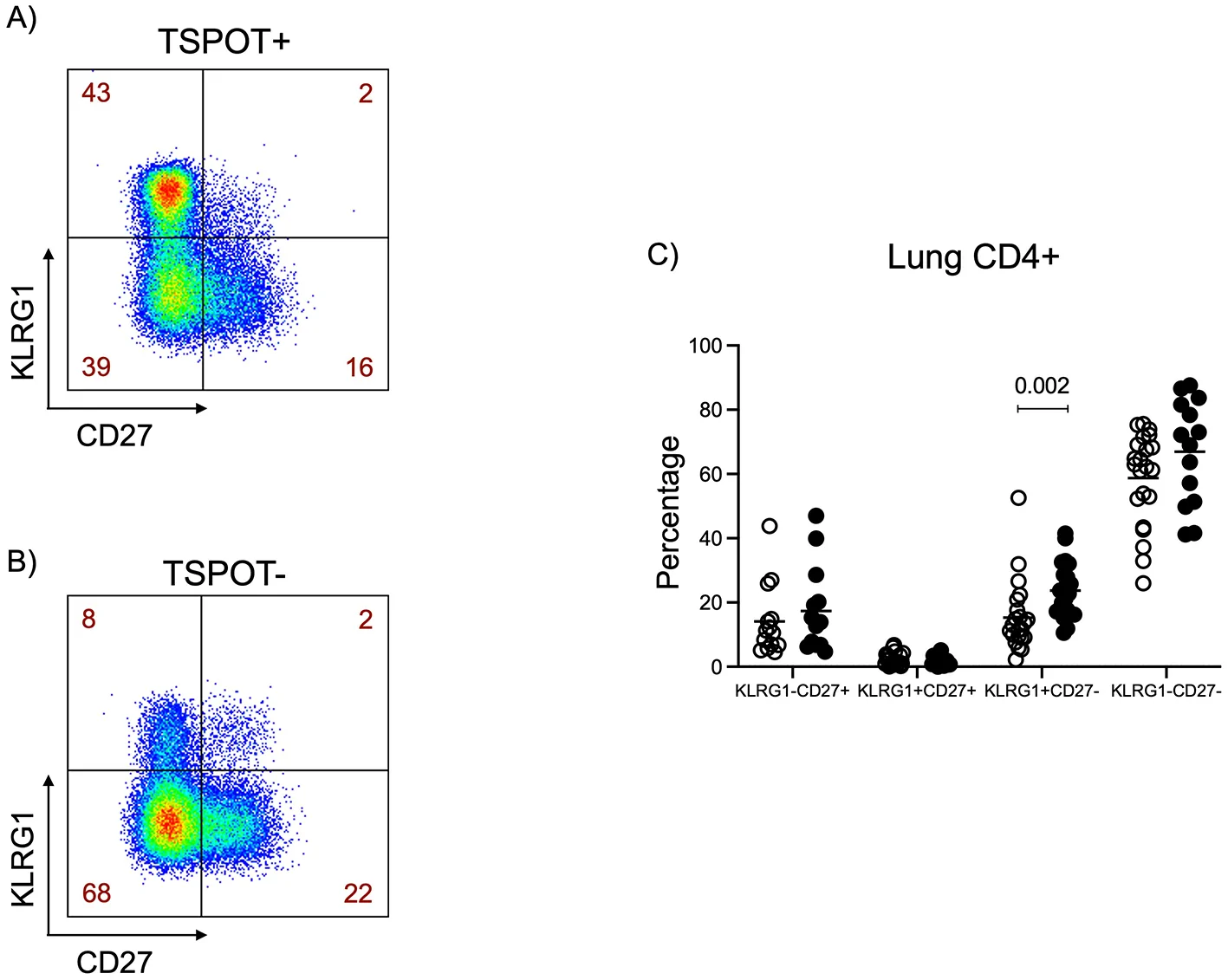
Higher expression of KLRG1 on CD27- CD4 T cells in the lungs of TSPOT positive individuals. KLRG1/CD27 gating was applied to CD4+ T cells from all tissues. Representative KLRG1/CD27 FlowJo plots of 27-year-old TSPOT+ female **(A)** and 43-year-old TSPOT– male **(B)** are shown. Data in figure **(C)** was from all subjects. The Mann Whitney test was used to compare medians between the two groups, with Bonferroni correction applied to account for multiple comparisons. Statistical significance was defined as a corrected *p* < 0.05, indicated by lines above each graph.

Finally, we observed higher expression of the marker KLRG1 on CD27- (mature) CD4 T cells in the lungs of TSPOT positive individuals. Representative plots illustrating this difference are shown in Figures 4A (TSPOT+) and 4B (TSPOT−), whereas Figure 4C presents aggregated data from all individuals, demonstrating the statistical significance of this observation, and suggesting a more differentiated T cell phenotype in response to previous *M.tb* infection. This pattern was not observed in CD8 T cells, or CD4 T cells from other tissues. These findings collectively highlight tissue-specific immune signatures associated with IGRA positivity.

## Discussion

This study aimed to explore whether the immune reactivity measured by the TSPOT.*TB* assay translates into differences in tissue-resident immune cell profiles between TSPOT positive and negative individuals. All participants were clinically healthy, and postmortem samples were taken from various lung sections, allowing MGIT bacterial culture to confirm the absence of ongoing *M.tb* infection. This was crucial since TSPOT positivity represents a spectrum from subclinical infection to incipient TB or even non-infectious immune memory.

The prevalence of IGRA positivity in our study population (48%) aligns with previous reports from Uganda (21), suggesting that our postmortem cohort has at least a similar prior exposure to other subjects in the general population. Our results demonstrate that the immune cell landscape, as measured by total CD3, CD4, CD8, and CD19 cell frequencies, were largely similar between TSPOT+ and TSPOT– individuals across all tissue types examined. There were similar CD45RA/CCR7-defined memory T cell subsets and IgD/CD27-defined memory B cell subsets between the two groups, across all tissues examined. This suggests that TSPOT positivity alone does not lead to major systemic alterations in these broad immune cell populations (22, 23).

We were particularly interested in finding out if there were differences in Tissue Resident Memory cells between the two groups. TRMs are known to play a critical role in lung immune surveillance and *M.tb* clearance (17, 24). We found higher frequencies of CD4+ TRMs in the lungs of TSPOT positive individuals compared to TSPOT negative individuals. Although the *M.tb* specificity of these cells was not assessed, their elevated presence in the lung tissue of TSPOT+ individuals suggests localized immune memory linked to prior *M.tb* exposure. This was not observed in CD8 or CD19 cells, nor in other tissues, underscoring the critical role of CD4 T cells in TB immunity and the localization of the immune response to the lungs, the primary site of TB infection.

In a TB endemic setting, *M.tb* is known for developing a chronic infection in the lungs, which is associated with T cell activation, differentiation, exhaustion, and senescence. Indeed, various studies have found higher levels of activation and exhaustion on T cells in active TB, which reduces with treatment (25, 26). This suggests an increase in cell activation and exhaustion with increasing disease. In our cohort, however, we report similar expression of CD27, KLRG1, CD57, and CD28 on overall CD4 and CD8 T cells across all tissues. Our analysis revealed higher HLA-DR expression on CD8 T cells in the lung of TSPOT positive individuals, but higher levels on CD4 T cells in the blood of TSPOT negative individuals. High HLA-DR expression has been associated with new *M.tb* Infection (27, 28). The fact that all tissues in both our TSPOT negative and positive individuals were MGIT negative suggests there was no recent infection with *M.tb*. However, we cannot rule out the possibility of early infection in a section of lung that was not sampled. It is unclear at this stage what the higher HLA-DR expression on CD8 T cells may mean.

The elevated expression of KLRG1, a marker of terminal differentiation and impaired proliferation (29–31), on mature CD4 CD27- T cells (32, 33) in the lungs of TSPOT+ individuals support the idea of a more differentiated and potentially exhausted immune phenotype in these individuals. Given that the study was conducted in a TB endemic setting, likely prolonged or repeated *M.tb* exposure could drive CD4 T cells to such a phenotype. High KLRG1 expression has been linked to reduced extravasation and diminished capacity for homing to parenchymal tissues, which resulted in reduced *M.tb* clearance (34). Altogether, our data suggests that TSPOT positivity might reflect not only immune memory, but also the presence of highly differentiated CD4 T cells in tissue-specific compartments, particularly the lungs, where *M.tb* infection is most concentrated.

## Conclusion

Our study demonstrates that IGRA positivity, while useful in identifying individuals with a recall response to *M.tb*, does not uniformly translate into widespread immune alterations across tissues. However, it is associated with localized immune changes, particularly in the lungs, where an increased presence of CD4 TRMs and terminally differentiated mature CD4 T cells, may reflect a state of heightened immune surveillance or chronic immune engagement following *M.tb* exposure. These findings raise questions as to whether these T cells contribute to disease progression under certain conditions, such as immunosuppression or TB re-exposure.

## Data Availability

The raw data supporting the conclusions of this article will be made available by the authors, without undue reservation.

## Supplementary material

Supplementary Figure 1 Gating strategy for flow cytometry of PBMCs. Representative plot of total cells. Total cells were first gated by FSA/SSA, then singlets isolated, followed by selection of live cells and then CD19 negative and CD19 positive cells. The dump gate, used to make the CD3 gates cleaner, consisted of CD56 and CD14. CD3 positives were then separated based on CD4 and CD8 expression. CD4, CD8, and CD19 gating was followed by specific staining as represented in the results. Representative plots from a 68-year-old female TSPOT− subject.

Supplementary Figure 2 Gating strategy for flow cytometry of tissues (Spleen, BAL, HLN, Lung, and DLN). Representative plot of total cells. Total cells were first gated by FSA/SSA, then singlets isolated, followed by selection of live cells and then CD19 negative and CD19 positive cells. The dump gate, used to make the CD3 gates cleaner, consisted of CD56 and CD14. CD3 positives were then separated based on CD4 and CD8 expression. CD4, CD8, and CD19 gating was followed by specific staining as represented in the results. Representative plots from a 68-year-old female TSPOT- subject.

Supplementary Figure 3 CD4 and CD8 Memory T cell subsets across tissues. CD4 **(A–D)** and CD8 **(E–H)** T cells were subdivided based on CCR7 and CD45RA expression into four subsets: Naïve **(A, E)**, Central Memory (TCM; **B, F**), Effector Memory **(C, G)**, and Terminally Differentiated Memory **(D, H)**. Data was from all subjects. The Mann Whitney test was used to compare medians between the two groups, with Bonferroni correction applied to account for multiple comparisons. Statistical significance was defined as a corrected *p*-value < 0.05. No significant differences were observed between the groups.

Supplementary Figure 4 Memory B cell subsets across tissues. IgD and CD27 expression were used to differentiate B cells into Naïve **(A)** (IgD+CD27), Memory B cells **(B)** (IgDCD27+), and Atypical memory **(C)** (IgDCD27). Data was from all subjects. The Mann Whitney test was used to compare medians between the two groups, with Bonferroni correction applied to account for multiple comparisons. Statistical significance was defined as a corrected *p*-value < 0.05. No significant differences were observed between the groups.
