# Supplementary material for: Tissue-specific T cell profiles in Mycobacterium tuberculosis uninfected IGRA negative and positive individuals
Source: Front Tuberc. 2025 Mar 6;3:1535945. doi: 10.3389/ftubr.2025.1535945 (PMC13494974; doi:10.3389/ftubr.2025.1535945)
Supplement: Supplementary file 1 [file Table_1.pdf]

Table 1. Patient Demographics and clinical characteristics

Demographic	Male (TSPOT+)[a]	Female (TSPOT+)	Total (TSPOT+)
Age range			
18-35	26 (10)	4 (2)	30 (12)
36-55	10 (5)	5 (3)	15 (8)
≥56	4 (4)	3 (1)	7 (5)
Cause of death			
Trauma			
Head trauma	30 (11)	7 (4)	37 (15)
Abdominal trauma	9 (8)	0 ()	9 (8)
Gunshot	1 (0)	0 ()	1 (0)
Non trauma			
Intestinal obstruction	0 (0)	2 (1)	2 (1)
Duodenal ulcers	0 (0)	3 (1)	3 (1)
Employment			
Skilled labourer (plumber, electrician, welder, mechanic, construction worker)	8 (4)	0	8 (4)
Military/police	7 (3)	1 (0)	8 (3)
Business person/owner	10 (5)	5 (3)	15 (8)
Farmer or animal husbandry	3 (1)	1 (1)	4 (2)
Health care worker	2 (1)	3 (1)	5 (2)
Bar or restaurant worker	6 (2)	2 (1)	8 (3)
Truckdriver/driver/conductor	2 (2)	0	2 (2)
Not employed	2 (1)	0	2 (1)
Smoking/substance use			
Tobacco	3 (3)	0	3 (3)
Other drug	4 (1)	0	4 (1)
Not known	2 (2)	4 (3)	6 (5)
None	31 (13)	8 (3)	39 (16)
Alcohol use			
Yes	18 (10)	4 (1)	22 (11)
No	22 (9)	8 (5)	30 (14)
Blood transfusion			

Yes	0 (0)	1 (0)	1 (0)
No	40 (19)	11 (6)	51 (25)
Time in hospital			
Death on arrival	8 (6)	2 (2)	10 (8)
<1day	26 (11)	10 (4)	36 (15)
1-7days	5 (2)	0	5 (2)
>7days	1 (0)	0	1 (0)

[a]Total in group (number that were TSPOT+ in group)
